# Aversive Learning and Trait Aggression Influence Retaliatory Behavior

**DOI:** 10.3389/fpsyg.2016.00833

**Published:** 2016-06-08

**Authors:** Tanaz Molapour, Björn Lindström, Andreas Olsson

**Affiliations:** ^1^Department of Clinical Neuroscience, Karolinska InstitutetStockholm, Sweden; ^2^Department of Economics, University of ZurichZürich, Switzerland

**Keywords:** fear-conditioning, learning, social, aversive, retaliation, aggression, anti-social, interaction

## Abstract

In two experiments (*n* = 35, *n* = 34), we used a modified fear-conditioning paradigm to investigate the role of aversive learning in retaliatory behavior in social context. Participants first completed an initial aversive learning phase in which the pairing of a neutral conditioned stimulus (CS; i.e., neutral face) with a naturally aversive unconditioned stimulus (US; electric shock) was learned. Then they were given an opportunity to interact (i.e., administer 0–2 shocks) with the same faces again, during a Test phase. In Experiment 2, we used the same paradigm with the addition of online trial-by-trial ratings (e.g., US expectancy and anger) to examine the role of aversive learning, anger, and the learned expectancy of receiving punishment more closely. Our results indicate that learned aversions influenced future retaliation in a social context. In both experiments, participants showed largest skin conductance responses (SCRs) to the faces paired with one or two shocks, demonstrating successful aversive learning. Importantly, participants administered more shocks to the faces paired with the most number of shocks when the opportunity was given during test. Also, our results revealed that aggressive traits (Buss and Perry Aggression scale) were associated with retaliation only toward CSs associated with aversive experiences. These two experiments show that aggressive traits, when paired with aversive learning experiences enhance the likelihood to act anti-socially toward others.

## Introduction

Our social interactions are largely determined by past and present learning about the people we are interacting with. One of the most influential ideas in psychology is that human social behavior is, to a large extent, governed by likes and dislikes formed about others (Allport, 1935; Martin and Levey, 1978). Indeed, people will increasingly prefer to spend time with individuals they learned to like, and try to avoid, or aggress toward those they learned to be afraid of or dislike. Although adaptive in some situations, anti-social behaviors, such as avoiding, aggressing, or punishing others, can be detrimental to interpersonal relationships. For example, an initial, small aversive encounter with a new neighbor might lead to avoidance or aggressions. These behaviors might, in turn, result in retaliatory behavior and, down the road, an escalating circle of self-reinforcing aggression. Given the pervasive impact of negative evaluations on behavior (Chen and Bargh, 1999; Scherer, 2005), it is important to understand how learned negative evaluations are formed and changed during social interactions. The role these learning processes play in the unfolding of aversive social interactions is largely unknown.

We used a simple experimental model of social interaction based on classical fear-conditioning to study the role of aversive learning in human social behavior. Here, retaliation/anti-social behavior was operationalized as the administration of shocks to a co-player in an alleged social interaction (see Section Methods; Miller and Eisenberg, 1988). We examined whether aversive learning about co-players could explain subsequent retributive and anti-social behavior.

Classical fear conditioning has been used experimentally to study the nature of phobias (Pavlov, 1927; Phelps and LeDoux, 2005), and responses to others in social situations (Olsson and Phelps, 2007; Molapour et al., 2015). Fear conditioning involves pairing a neutral conditioned stimulus (CS; e.g., neutral face) with a naturally aversive unconditioned stimulus (US; e.g., electric shock). After sufficient pairings, presentations of the CS alone come to elicit conditioned skin conductance responses, SCR (Phelps and LeDoux, 2005). Failing to form and/or express the CS-UCS association may indicate a lack of anxiety or anticipatory fear, which in turn may give rise to antisocial acts in some individuals. Although deficits in fear conditioning have been found in adult psychopaths and criminals (Hare and Quinn, 1971; Flor et al., 2002; Birbaumer et al., 2005), little is known about how the development of conditioning is related to aggressive behavior in a social interactive context in the normal population. In a non-social context, research has shown that learned aversions may be the origin of common maladaptive behaviors, such as anxiety disorders (Mineka and Zinbarg, 2006; Dunsmoor et al., 2011) and phobias (Öhman and Mineka, 2001). However, there has also been suggestions that learned aversions are important for social valuations, and ostracism (Seymour et al., 2007; Molapour et al., 2015). Yet, few, if any, studies have investigated how learning mechanisms known to underlie learned aversions through Pavlovian conditioning, can help to explain interactive behavior, specifically anti-social behavior, in the normal population. Given the prevalence of anti-social behavior in everyday life (Folger and Baron, 1996; Cowie et al., 2002), understanding the underlying mechanisms is crucial, both to illuminate the dark side of human psychology, and in extension, inform preventive strategies.

There are several reasons for predicting that aversive learning might result in retaliatory behavior. For example, it has previously been suggested that stimuli associated with aversions through conditioning can enhance aggression. In support of this conjecture, research in rats has shown that conditioned stimuli increased the probability of fighting behavior when given the opportunity (Hutchinson et al., 1971). Similar to this finding, a limited number of experiments with humans also suggest that aversive associations can increase the intensity of aggression. For example, one study (Fraczek, 1974) showed that painful experiences associated with a specific colored stimulus incited stronger aggression to a peer. Specifically, it was shown that the presentation of a particularly colored shock delivering apparatus previously associated with pain, as compared to a different colored shock delivering apparatus not associated with pain, resulted in the administration of shocks with a longer duration toward the peer. This observation is consistent with aversive learning as the underlying mechanism (Fraczek, 1974). Although Fraczeks' paradigm reveals the importance of aversive learning in aggressive behavior, one limitation is that participants were required to punish the peer for incorrect answers, which could mean that aggression displayed by the subjects was an expression of wanting to help the confederate to improve performance on the task (Taylor, 1967; Baron and Eggleston, 1972; Rule and Nesdale, 1974) rather than expression of aggressive behavior. Furthermore, punishment has been extensively studied in economic public good games. However, in many natural interactions punishments work differently than the ways studied in economic public good games (Ostrom et al., 1992; Fehr and Gächter, 2002; Wu et al., 2009). For example, in these paradigms monetary loss is used as a social punisher. In our paradigm, we wanted to bypass such economic motivators. Instead, we used shocks to model the effects of primary (naturally aversive) punishers to understand the basic processes underlying retaliation. We reasoned that this approach would facilitate the generalizability of our results beyond the economic domain.

A variety of other aggression paradigms, not explicitly studying aversive learning, have been used to understand different factors influencing aggressive behavior in general (See Review, Anderson and Bushman, 2002). In these aggression paradigms participants can typically act aggressively/anti-socially toward others, by, for example, administering shocks or hot sauce. These paradigms reveal that although people are reluctant to act aggressively toward others in general, some motivational factors, such as competiveness, provocation, and aggressive cues (e.g., guns) can influence participants to aggress more often, suggesting aggressive behavior toward others in certain circumstances (Anderson and Bushman, 2002). These are often referred to as situational factors. Characteristics of the agent (e.g., personality traits, attitudes, genetic predispositions) are also important factors in contributing to aggressive behavior (Mischel and Shoda, 1995). Aggression theories have also implicated that general arousal and aggressive cues combine to increase aggression (Berkowitz and Lepage, 1967; Bandura, 1973). That is to say, if an angered person's arousal is attributed to the frustrating or insulting source of the anger, subsequent aggression will increase (Rule and Nesdale, 1976). In contrast to how fear motivates escape and finding safety (Frijda, 1986; Lazarus, 1991; Levenson, 1999; Öhman and Mineka, 2001); anger, the response to a personal transgression, motivates aggression that stops ongoing, and/or deters future, transgressions (Frijda, 1986; Lazarus, 1991; Levenson, 1999). Other studies have shown that emotions such as anger motivate individuals to punish opportunistic behavior. Unkind or selfish behavior induces anger and the angrier people are, the more likely they are to incur costs to penalize such behaviors (Bosman and Van Winden, 2002; de Quervain et al., 2004).

The studies outlined here indicate that both conditioned and unconditioned aversive stimuli in humans and animals that are associated with unpleasant events can produce aggressive responses even though they were not, by themselves, directly responsible for any aversive incidents (e.g., Hutchinson et al., 1971; Fraczek, 1974).

This highlights the possible importance of aversive learning in aggressive behavior, however, the role it plays in social interactive context, in which participants can freely choose how to interact, is unknown. Furthermore, previous studies demonstrate that factors, such as expectancy of punishment, arousal, trait and state anger, can increase general aggression in social contexts. Our aim was to investigate the interaction between these constructs to better understand anti-social behaviors in social contexts.

### The present study

In order to elucidate the role of learning in retaliatory behavior we monitored aversive learning in a simple interactive situation in which participants could receive and reciprocate shocks to alleged co-players. Retaliatory behavior was operationalized as the administrations of shocks to co-players. Aversive learning was measured through the skin conductance response (SCR), and online ratings of shock expectancy and anger were used to assess explicit evaluations. We hypothesized that if learned aversions would influence retaliatory behavior, participants would administer more shocks to the faces that were previously paired with most number of shocks (i.e., CS++, and CS+, see Section Methods). In Experiment 2, we used the same paradigm with the addition of online trial-by-trial ratings (e.g., US expectancy and anger) to examine the role of aversive learning and emotions (i.e., US expectancy and anger) in retaliatory behavior. We expected to find that aversive learning, arousal, anger and US expectancy, each contribute to our measure of anti-social behavior.

## Methods

### Methods experiment 1

#### Participants

Thirty-eight (23 women), healthy normal volunteers ranging from 18 to 29 years old (*M* = 22.76, *SD* = 3.10) were recruited. Participants (*n* = 3) that reported not receiving shocks to CS+ and CS++ were excluded from all analysis (sample; 35, 21 women). For the SCR analysis only, participants (*n* = 12) who did not show larger SCRs to at least one of the CS+'s compared to CS− were excluded, resulting in a sample of 23 participants (14 Females) (age; *M* = 22.57, *SD* = 3.33). Participants received two movie vouchers for their participation. All participants gave informed consent before participation and were naive to the purpose of the experiment. The procedures were executed in compliance with relevant laws and institutional guidelines, and were approved by the Regional Ethical Review Board of Stockholm.

#### Stimuli and apparatus

The CSs consisted of three pictures of Caucasian male faces with neutral facial expressions, from the NimStim Set of Facial Expressions (Models: 23, 28, 36; Tottenham et al., 2009). Electrical stimulation was delivered through a pair of Ag electrodes of 20 × 25 mm with a fixed interelectrode mid-distance of 45 mm. Shock deliverance was controlled by a monopolar DC-pulse electric stimulation (STM200; Biopac Systems Inc., www.biopac.com). Between the electrodes and the skin, a conductive gel (Signa, Parker) was applied. For SCR assessment two Ag/AgCl electrodes of 20 × 16 mm were attached with to the medial phalanges of the first and third fingers of the non-preferred hand. The physiological signals were amplified and recorded using BIOPAC Systems (Santa Barbara, CA) hardware at a rate of 250 samples per second. Data were analyzed using AcqKnowledge software (BIOPAC Systems). Participants sat in front of a standard 21-inch cathode ray tube (CRT1) monitor (100 cm distance) in a sound attenuated chamber. The resolution of the screen was 800 × 600 pixels with a refresh rate of 60 Hz. The experiment was presented in E-Prime 2.0 (Schneider et al., 2002).

#### Subjective assessments

Evaluation of the US was assessed on an 11-point scale ranging from −5 (unpleasant) to 5 (pleasant). General level of anxiety was measured with the Trait Anxiety Inventory (STAI-T; Spielberger et al., 1970). Alpha internal consistency of the STAI-T is 0.91 (Spielberger et al., 1970). The Buss-Perry Aggression scale (Buss and Perry, 1992) was employed to measure trait aggression through likert scale based answers ranging from “extremely uncharacteristic of me” to “extremely characteristic of me” on 29 items. These measure four subscales, namely physical aggression, verbal aggression, anger, and hostility. The internal consistency for each factor is as followed: Physical Aggression, 0.85; Verbal Aggression, 0.72; Anger, 0.83; and Hostility, 0.77 (total score = 0.89; Buss and Perry, 1992). Participants also completed the balanced emotional empathy scale (BEES; Mehrabian, 1996). Alpha internal consistency of the BEES was 0.87 (Mehrabian, 1997).

#### The cover story

Participants were instructed that three other co-players were participating in the study at the same time. The participants were told that all four of them would interact through our online network. In fact, no other co-players existed and the actions of the “co-players” were controlled by a computer program. To ensure that the participants believed our instructions, they were told they would not meet the other co-players face-to-face to ensure their anonymity. Participants were told that throughout different parts of the experiment they would be able to administer or receive shocks to-and-from the other co-players. Participants were specifically told that only the person that “randomly” was selected to start (i.e., always the co-player) could administer shocks during the first part of the experiment. To increase credibility of the cover story, a picture was taken of each participant, and they were told their picture would be edited and added to the computer program. They were also informed that the computer program worked in such a way that the image of the person who appeared on the screen indicated that the same person was viewing a picture of them at the same time. That is, if they received a shock, it would be the person whose image was on the screen that was administering the shock. Similarly, if the participant decided to give a shock, their image would be presented on the screen of their co-player receiving it. No information was given about the specific face-shock contingencies; meaning that the participants received no information that one face was paired with two shocks, another face with one shock, and one face with no shocks. Participants were also told that the computer would “randomly” choose which part of the experiment he/she would start with, which would imply who would be able to give/receive shocks first. The participant always started with an Acquisition phase (participants could not administer any shocks during this phase), followed by a final Test phase (participants could administer shocks during this phase).

### Procedure

#### Acquisition phase

**Figure 1:**
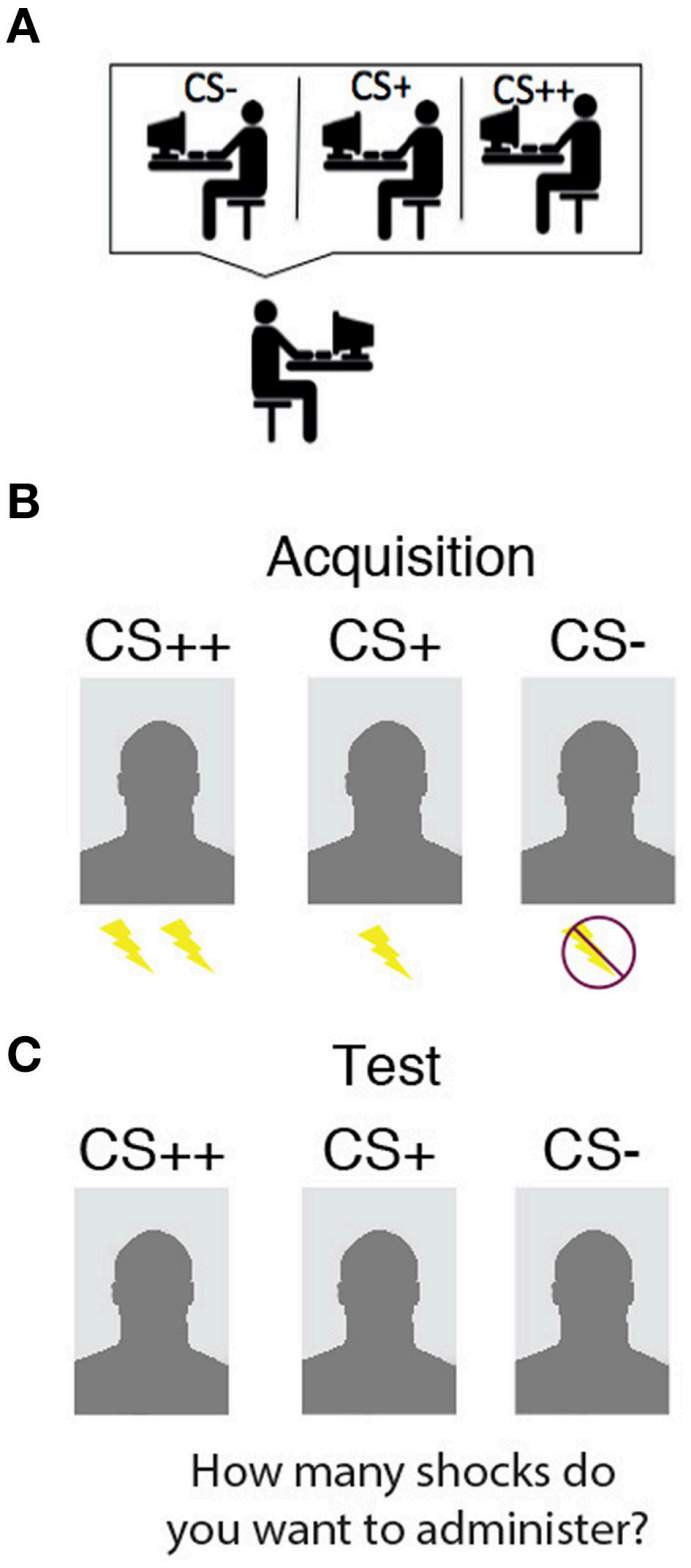
**Task design. (A)** Participants were told they are interacting with three other co-players. **(B)** During Acquisition, each one of the three co-players represented the CS++, paired with two shocks when displayed; CS+, paired with one shock when displayed; and CS−, never paired with a shock. **(C)** During the Test phase, participants watched all CSs again, and were asked how many shocks they wanted to administer.

The SCR, and shock electrodes were attached and US intensity level was determined by gradually increasing shock intensity until participants indicated the shock to be “uncomfortable though not painful.” All participants underwent fear conditioning during the first part (Acquisition phase). The CSs consisted of three different images (male faces with neutral facial expressions), which were paired with mild shocks to the wrist (US) with a reinforcement rate of 75%. The CS++ was paired with two consecutive shocks (each 100 ms), the CS+ was paired with one shock, while the CS− was never paired with a shock (see Figure 1). All CSs were presented six times for 9 s. Fear conditioning consisted of 18 trials in total. Assignment of the pictures as CS++, CS+, or CS− was counterbalanced across participants. CSs were presented on a white background in frontal perspective. Stimulus presentation was pseudo-randomized to prevent the occurrence of two consecutive trials of the same CS type (CS++, CS+, or CS−) throughout the experiment. A black fixation cross on a white background was shown during inter-trial intervals (ITIs), which lasted between 8.0 and 12.0 s (*M* = 10.0 s).

#### Test phase

After the Acquisition phase, participants were instructed that the Test phase would be the last part of the experiment and that they were now able to administer shocks to the other co-players. CSs were presented equal number of times as in the Acquisition phase. The CSs were presented for approximately same duration (i.e., 5.0–7.0 s) before a shock could be administered, similar to when a shock could be received during the Acquisition phase. For each CS presentation, participants were asked to indicate (5.0 s after onset of CS) how many shocks they wanted to administer to the person displayed on the screen. The CSs were displayed for the whole duration (9 s) regardless of the decision. Participants could choose to give 0–2 shocks. Participants indicated their responses using three keys that were marked with the numbers (0, 1, 2) on the keyboard. Participants themselves did not receive any shocks during this phase. At the end of the Test phase participants filled out questionnaires and ratings of each CS, completed four different questionnaires (STAI-S, Buss Perry, and BEES) and a debriefing form.

#### Statistical analyses

Number of administered shocks, SCR, and US expectancy ratings were subjected to analyses of variance (ANOVAs) with stimulus (CS++, CS+, and CS−) as the within-subjects factor. To establish the *strength* of administration of shocks, we calculated the difference score of CS+s (*d*-score = administered shocks to CS++ minus administered shocks to CS+) to use it in the correlation analysis. SCRs to the CSs were calculated by measuring the largest response 0.5–4.5 after stimulus onset for each CS trial. SCR responses below 0.02 microSiemens (μS) were recorded as zero. Raw SCRs were square root transformed to normalize the distributions, and scaled according to each participant's mean square-root-transformed US response. Participants that did not show larger SCRs to at least one of the CS+s as compared to CS− were excluded from the analysis. A repeated-measures analysis of variance (RM ANOVA) was used to compare SCRs. The alpha level was set at 0.05 for statistical analyses. A Greenhouse–Geisser procedure was used in case of violation of the sphericity assumption in the ANOVAs. We also conducted non-parametric test (see Supplementary Materials). We also assessed, Anger online ratings, Buss Perry, as predictors of administered shocks, SCR, and US expectancy through correlation analysis. We also conducted a mediation analysis to examine whether the predictors (anger and trait aggression) accounted for administration of shocks. The relationships between anger, trait physical aggression, and administration of shocks, were first analyzed through Pearson correlations. Mediation analysis (see Baron and Kenny, 1986) was then carried out to statistically determine whether the effect anger on administration of shocks was explained by physical trait aggression. We then performed a Sobel test (1982) to ensure that the indirect effect of the IV on the DV via the mediator was significantly different from zero. Mediation coefficients and Sobel test statistics were obtained using the SPSS macros and procedures developed by Preacher and Hayes (2004).

### Results experiment 1

#### Skin conductance response

We examined if learning, as indexed by skin conductance, occurred during Acquisition. Comparing SCR responses to the different CSs we found that participants showed largest SCR responses to CS++ (i.e., the CS paired with 2 shocks) and CS+ (i.e., the CS paired with 1 shock), as compared to CS− (i.e., the CS not paired with shock). We measured SCR responses to CS++, CS+ and CS− in a repeated-measures (RM) analysis of variance (ANOVA) with Stimulus (CS++, CS+, CS−) as within-subjects variable. Although, our analysis did not reveal a main effect of CS [*F*_(1.56, 34.31)_ = 2.31, *p* = 0.125, η^2^ = 0.095], pairwise comparisons revealed significant difference between CS+ and CS− (*p* = 0.033), and a trend between CS++ vs. CS− (*p* = 0.053), however, there was no significant difference between CS++ vs. CS+ (*p* = 0.80).

#### Administered shocks to CSs

**Figure 2:**
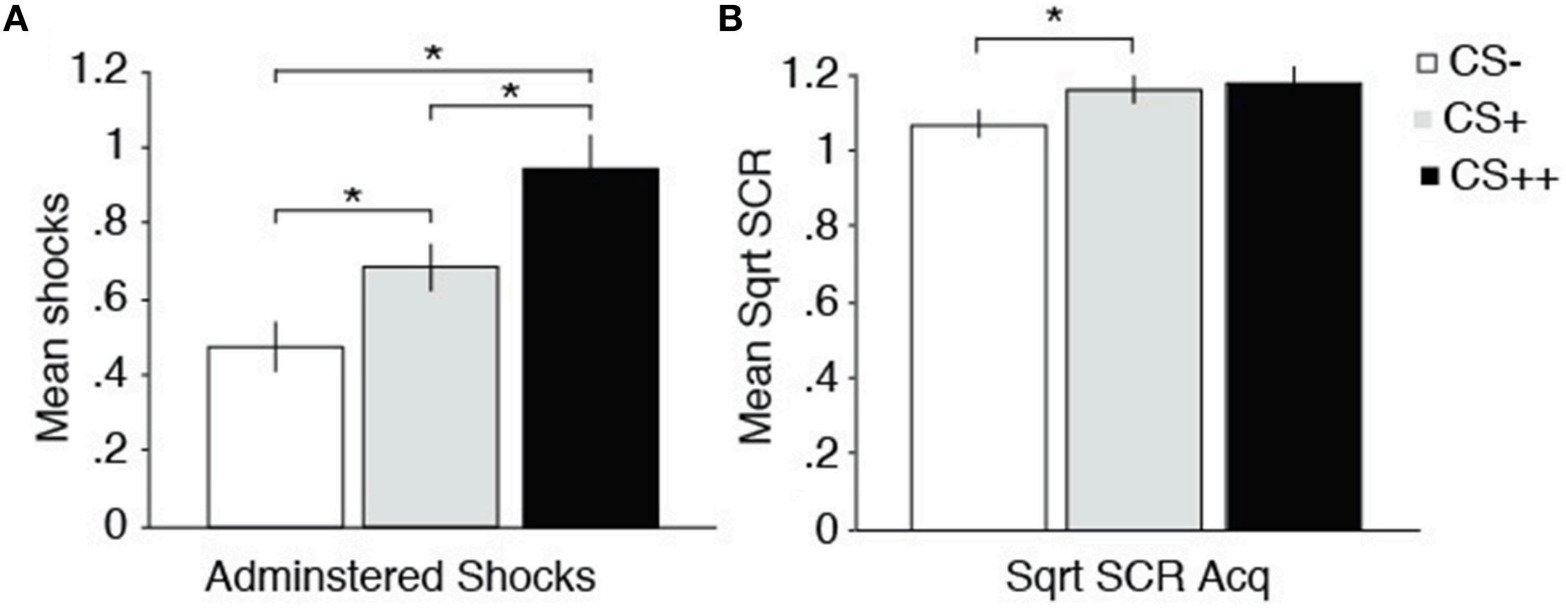
**Administration of shocks and SCR results**. **(A)** Average number of administered shocks to each CS during the Test phase, showing a linear increase in punishing behavior as a function of received shocks. **(B)** The amplitude of SCRs is shown in microSiemens, showing stronger SCRs CS+ relative to CS− during Acquisition. Error bars indicate SEM. Asterisks indicate a statistically significant differences *p* < 0.05.

In order to investigate whether learned aversions influenced retaliatory behavior we examined how many shocks were administered to the different CSs during the Test phase. Using the whole sample (*n* = 33), we found that participants chose to give more shocks to CS++, as compared to CS+ and CS− [main effect of CS; *F*_(1.69, 54.05)_ = 13.77, *p* = < 0.0001, η^2^ = 0.30; See Figure 2]. Pairwise comparisons revealed significant differences between CS++ vs. CS− (*p* < 0.0001), CS+ vs. CS− (*p* = 0.022), and between CS++ vs. CS+ (*p* = 0.001). Finally, to investigate the relationship between SCRs and administration of shocks we performed a correlation analysis which revealed no significant relationship between administration of shocks and SCRs [*r*_(23)_ = 0.40*, p* = 0.062] to CS+'s (see Table 1).

**Table 1:** **Pearson Correlations**.

	**BPAS verbal aggression**	**BPAS anger**	**BPAS hostility**	**BPAS physical agg**	**STAI-T**	**BEES**	**Anger Online rating**	**US expectancy rating**
Shocks	0.256	−0.001	0.039	0.492^**^	−0.169	−0.355^*^	0.389^*^	0.469^**^
BPAS verbal aggression		0.476^**^	0.306	0.450^*^	−0.275	−0.306	0.032	0.117
BPAS anger			−0.019	0.246	−0.153	0.084	0.186	0.188
BPAS hostility				0.001	0.651^**^	−0.058	0.036	−0.087
BPAS physical agg					−0.282	−0.586^**^	0.437^*^	0.409^*^
STAI-T						0.122	0.174	−0.085
BEES							−0.038	−0.295
Anger online rating								0.478^**^

** *Correlation is significant at the 0.01 level (2-tailed)*.

* *Correlation is significant at the 0.05 level (2-tailed). N = 31*.

*Shocks is mean number of administration of shocks to CS++ (minus) CS+*.

*BPAS Verbal aggression, Anger, Hostility, physical aggression are the different subscales in the Buss-Perry Aggression scale*.

*STAI-T is a questionnaire assessing general anxiety*.

*BEES is the balanced emotional empathy scale -Anger online rating is a trial-by-trial anger rating towards the CS++ (minus) CS+*.

#### Discussion experiment 1

Our results indicate that participants took the opportunity to retaliate against the co-players, who had given them shocks during Acquisition. The number of shocks the participants administered were proportional to how many shocks the CS was paired with during Acquisition. However, investigating the relationship between SCR responses and administration of shocks, we found no significant relationship between the two. That is, higher arousal did not predict administration of more shocks to the CSs, which is in contrast to previous studies showing that higher general arousal and aggressive cues together increase aggression (Baron, 1971; Bandura, 1973; Berkowitz, 1993). Our results did not allow us to conclude why the number of shocks predicted the retaliatory behavioral effects. To address the question about possible psychological mechanisms causally linking learning to retaliatory behavior, we conducted another experiment. Experiment 2 was first intended to replicate the results from Experiment 2. Importantly, we added self-reported measures of expectations of shocks and anger toward the CS investigate the role of expectations of punishment (e.g., US expectancy) and anger (e.g., trait and situational) in linking aversive learning and anti-social behavior (e.g., Bosman and Van Winden, 2002; de Quervain et al., 2004). Additionally, participants were given the option to “remove” the CS (i.e., replacing image of the face with a white screen), in order to capture more avoidance-motivated behavior in contrast to giving 0, 1, or 2 shocks. Based on theories disentangling decisions to escape or aggress based on emotions such as fear or anger (Frijda, 1986; Lazarus, 1991; Levenson, 1999; Öhman and Mineka, 2001) we hypothesized that our new additional measures (e.g., US expectancy and Anger ratings) would help to further explain the link between aversive learning and anti-social behaviors that we observed in Experiment 1.

### Methods experiment 2

#### Participants

In Experiment 2, 34 (18 women) new, healthy, students ranging from 18 to 35 years old (*M* = 26.03, *SD* = 4.88) were recruited. For the SCR analysis, we excluded participants who did not show larger SCRs to at least one of the CS+'s as compared to the CS− (*n* = 5). For all other analyses, we excluded participants who did not report larger US expectancy to at least one of the CS+'s as compared to the CS− (*n* = 3), resulting in a final sample of 31 participants (17 women) (age; *M* = 26.00, *SD* = 5.00).

#### Online US expectancy ratings

To assess participants' expectancy of punishment from the CSs, we measured US expectancy online during each image presentation on a 9-point scale ranging from 1 (certainly no electric stimulus) through 5 (uncertain) to 9 (certainly an electric stimulus). The scale was placed at the top of the screen above the CS picture. Participants rated US expectancy levels by using the numbers on the keyboard by pressing a number key representing the rating within 7 s following CS onset.

#### Online anger ratings

To examine whether anger could predict anti-social behavior during the Test phase, we collected anger ratings online after each image presentation, on a 9-point scale ranging from 1 (not angry at all) through 5 (uncertain) to 9 (very angry) during Acquisition. The scale was placed in the middle of the screen. Participants rated how angry they felt (i.e., “How angry do you feel with the person you just saw?”) after each CS presentation by using the numbers on the keyboard by pressing a number key representing the rating.

### Procedure

#### Acquisition phase

All procedures during Acquisition were identical to Experiment 1, except that in Experiment 2, participants also rated their US expectancy and Anger online rating during and after each image presentation.

#### Test phase

All procedures during Test were identical to Experiment 1, except that in Experiment 2, in order to capture avoidance behavior, in addition to giving (0–2) shocks, participants could also choose the option of not giving a shock and removing the image (by selecting X) of the co-player (i.e., the CS) (replacing it with a white screen for the remaining time). Participants indicated their responses using four keys that were marked with the symbols (0, X, 1, 2) on the keyboard. Participants were told explicitly that the duration of experiment would be exactly the same regardless of their choices.

### Results experiment 2

#### Skin conductance response acquisition

To assure that learning did occur during the Acquisition phase, we measured SCR responses to CS++, CS+, and CS− in a RM ANOVA with Stimulus (CS++,CS+, CS−) as within-subjects variable. This analysis revealed successful CS++/CS+/CS− differentiation in SCRs to the CSs, as indicated by a significant main effect of stimulus, [*F*_(2, 56)_ = 11.45, *p* < 0.0001, η^2^ = 0.29]. Pairwise comparisons revealed significant differences between CS++ vs. CS− (*p* = 0.005), and in contrast to Experiment 1, CS+ vs. CS− (*p* < 0.00031) also revealed significant differences. Again, there was no significant difference between CS++ vs. CS+ (*p* = 0.093). The larger SCR response to CS+'s as compared to CS− indicates that participants learned to differentiate between the CSs paired with shocks and CS not paired with shock.

#### Administered shocks to CSs

**Figure 3:**
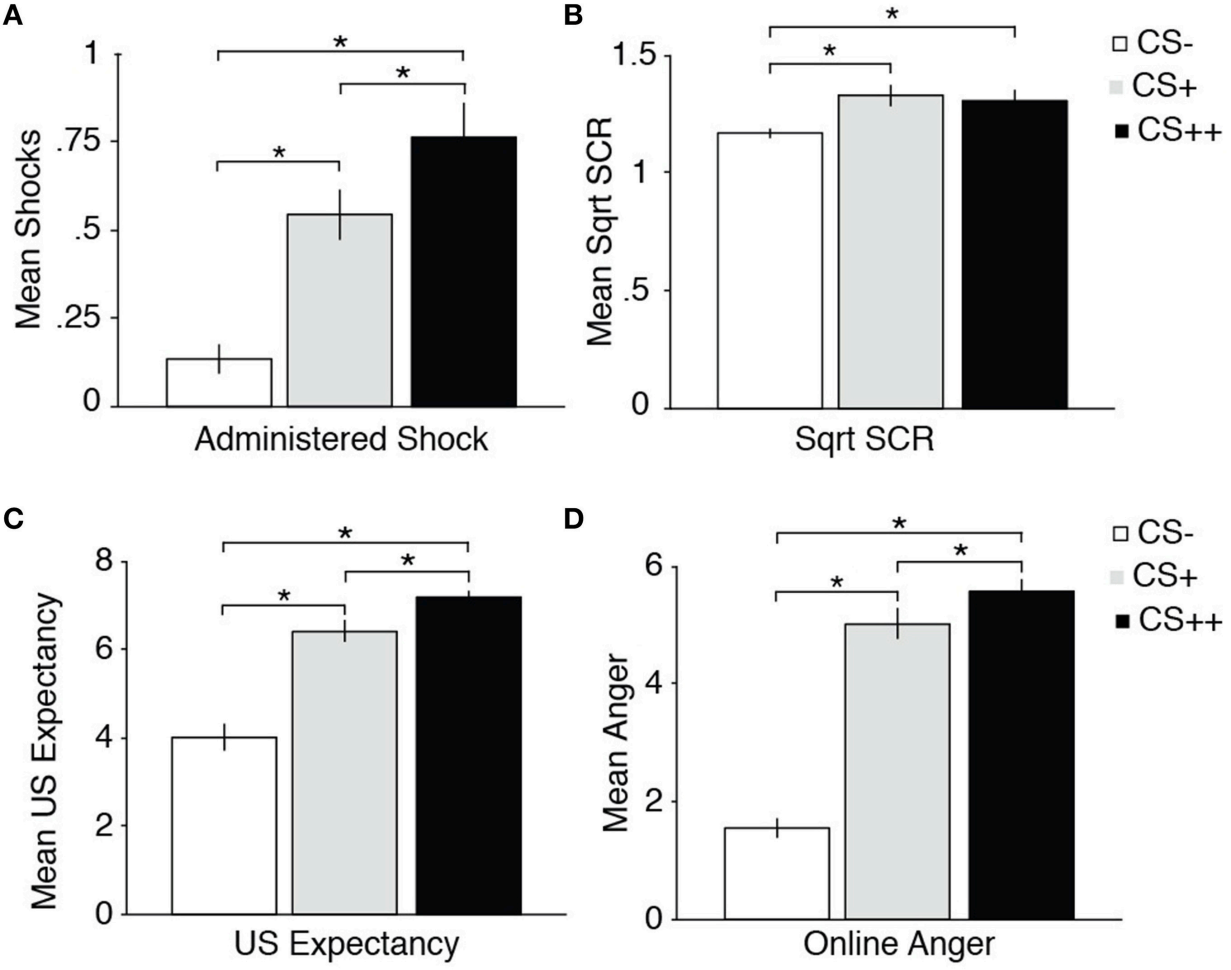
**Administration of Shocks, SCR, US Expectancy and Anger results**. **(A)** Average number of administered shocks to each CS during the Test phase, showing a linear increase in punishing behavior as a function of received shocks. **(B)** The amplitude of SCRs is shown in microSiemens, showing stronger SCRs to CS++, CS+ relative to CS− during Acquisition. **(C)** Mean US Expectancy ratings to CS++, CS+, and CS− during Acquisition. **(D)** Mean online Anger ratings to CS++, CS+, and CS− during Acquisition. Error bars indicate standard error of the mean (SEM). Asterisks indicate a statistically significant differences *p* < 0.05.

As in Experiment 1, we investigated whether learned aversions influenced anti-social behavior by examining how many shocks were administered to the different CSs during Test phase. Replicating the findings of Experiment 1, we observed a significant difference between the number of shocks administered to CS++, CS+, and CS− [main effect of CS; *F*_(2, 60)_ = 25.39, *p* < 0.001, η^2^ = 0.46; See Figure 3]. Follow-up paired samples *t*-test of CS− vs. CS++ revealed (*p* < 0.00001), CS− vs. CS+ (*p* < 0.00001), and CS+ vs. CS++ (*p* = 0.025). This again corroborates the findings from Experiment 1, that anti-social behavior is directly influenced by learned aversions during Acquisition.

#### US expectancy ratings

To assess the expectancy to receive a shock to the different CSs, we examined differentiation of US Expectancy between CS++, CS+, and CS− in a RM ANOVA with Stimulus (CS++, CS+, CS−) as within-subjects variable. In line with the skin conductance results, this analysis revealed successful CS++/CS+/CS− differentiation in reporting US expectancy, reflecting successful learning. We found a main effect of CS, [*F*_(1.63, 48.84)_ = 54.20, *p* < 0.001, η^2^ = 0.64]. Follow-up paired wise comparisons of CS− vs. CS++ revealed (*p* < 0.001), CS− vs. CS+ revealed (*p* < 0.001), and CS+ vs. CS++ revealed (*p* = 0.003).

#### Anger ratings

Based on previous studies showing that anger plays an important role in aggressive behavior (Frijda, 1986; Lazarus, 1991; Levenson, 1999), we used the online trial-by-trial anger ratings toward each CS type (i.e., CS−, CS+, CS++) to investigate the role of anger in aversive learning and anti-social behavior. Anger ratings were assessed using a RM ANOVA with Anger toward CSs (CS++, CS+, CS−) as a within-subjects variable. This analysis revealed significant anger ratings differentiating between CS++/CS+/CS−, as indicated by a significant main effect of CS, *F*_(2, 60)_ = 172.62, *p* < 0.001, η^2^ = 0.85. Follow-up paired samples *t*-test of CS− vs. CS+ revealed significant difference (*p* < 0.001), similarly, CS− vs. CS++ revealed a significant difference (*p* < 0.001), and so did CS+ vs. CS++ (*p* = 0.008). That is, participants felt proportionally angrier with CS++ as compared to CS+ and CS− during the Acquisition phase.

#### Factors influencing anti-social behavior

To explore if other possible factors were associated with anti-social behavior toward CSs we used online ratings and post-questionnaires in a subsequent correlation analysis. Our analysis revealed that physical trait aggression, as measured by Buss and Perry's (1992) physical aggression scale, correlated positively with the administration of shocks, suggesting that greater physical aggression traits were associated with anti-social behaviors only toward CSs associated with aversive experiences [*r*_(31)_ = 0.49, *p* < 0.05] to CS+'s (see Table 1). Further, analysis also revealed positive correlation between BusPerry physical aggression trait and anger online ratings [*r*_(31)_ = 0.44, *p* < 0.05] and US expectancy ratings [*r*_(31)_ = 0.41, *p* < 0.05] to the CS+s. Further, a mediation analysis revealed a significant indirect effect of trial-by-trial anger rating on number of shocks that were administered to the CS+s (β = 0.22, *p* = 0.031), mediated by physical trait aggression (Buss and Perry, 1992). After controlling for physical trait aggression, the initial significant relationship between anger trial-by-trial rating and number of administered shocks became non-significant (β = 0.40, *p* = 0.085). This indicates that level of personality trait aggression mediates the relationship between the anger towards the CS+s (co-players) and the administration of shocks to the CS+s in our experimental setting.

#### Post-experimental interviews

A post-experimental interview in Experiment 2 was used to assess participant's beliefs about the cover story and the motivation behind the experiment. Overall, most participants reported retributive motives when administering shocks: 75% (27 out of 36) of the participants reported retributive motives (e.g., “I wanted to teach the other person a lesson”) when administering shocks, others reported that they did it for fun 6% (2 out of 36), send a “message” to the other person 8% (3 out of 36), didn't know 8% (3 out of 36), and for the appearance of the other person 3% (1 out of 36). None of the subjects reported that they gave shocks because they thought the experiment or the researcher demanded it. Furthermore, in the post-experimental questionnaires 56% of the participants reported to have believed that they were receiving shocks from real participants, and 78% of participants reported that they believed that the other person was actually receiving shocks that they were administering. In Experiment 2, 61.3% believed that they were receiving shocks from real participants and 55.9 % believed that the other person was actually receiving shocks that they were administering.

#### Discussion experiment 2

In Experiment 2, we replicated the findings of Experiment 1, showing that participants seized the opportunity to retaliate against the co-players, who had given them shocks during Acquisition. Similar to Experiment 1, the number of shocks the participants administered was proportional to how many shocks the CS was paired with during Acquisition. Additionally, the added online trial-by-trial US expectancy ratings investigating the role of learned expectancy of receiving punishment corroborated the SCR findings, revealing larger expectancy responses to the CS+ and CS++ as compared to CS−. This indicated that participants learned to differentiate between the CSs paired with shocks and the CS not paired with shock. Furthermore, the additional online trial-by-trial anger ratings revealed that participants felt proportionally angrier with CS++, as compared to CS+ and CS−, during the Acquisition phase. In line with the hypothesis that personality traits contribute to aggressive behavior (e.g., Mischel and Shoda, 1995), our results show that physical trait aggression was associated with retaliation only toward CSs paired with shocks. Similar to Experiment 1, we did not find associations between SCR and retaliatory behavior in Experiment 2. However, we found a positive correlation between expectancy to receive shocks to CS+'s (CS++ minus CS+) and number of administered shocks to the CS+'s (CS++ minus CS+). These findings demonstrate that in addition to aversive learning influencing retaliatory behavior, aggressive traits, and expectancy of receiving punishment (i.e., US expectancy) enhance retaliatory behaviors.

## General discussion

The primary aim of the present investigation was to examine the role of aversive learning in retaliatory behavior. We used classical fear conditioning with an added Test phase allowing for social interactive behavior. This provided the opportunity to examine the strength of aversive learning about specific individuals and its influence on subsequent social interactive behavior with the same individuals.

In two separate experiments, we demonstrate how previously learned aversions influence future retaliatory behavior. In both experiments, participants showed largest SCRs to the faces paired with one or two shocks during Acquisition, demonstrating successful aversive learning. These findings were corroborated with US Expectancy ratings in Experiment 2. Critically, we demonstrated that participants administered more shocks to the individuals delivering the most number of shocks when the opportunity was given during the subsequent Test phase. Our findings are consistent with results on evaluative conditioning, showing that repeated pairings of CSs and USs influence subsequent evaluative *judgments* of the CSs (De Houwer et al., 2001; Baeyens et al., 2005; Walther et al., 2005), and classical fear conditioning studies showing that learned fear associations influence behavior (e.g., pathogenesis of anxiety disorders; Mineka and Zinbarg, 2006). Our results go beyond these findings and show that pairings of CS and aversive US enhanced retaliatory *behavior* toward another person in a social context.

We did, however, not find a significant effect of the individual participant's physiological arousal on retaliatory behavior. This finding stands in contrast to previous research showing that general arousal and aggressive cues combine to increase aggression (Berkowitz and Lepage, 1967; Bandura, 1973). One possible reason for this discrepancy is that factors other than physiological arousal (e.g., anger) during aversive learning motivated the participants to administer shocks to the co-players during the Test phase. Anger is a key motivator of aggression and has been linked to action tendencies related to “moving against” others (e.g., assaulting, attacking, kicking; Rule and Nesdale, 1976; Frijda et al., 1989; Roseman et al., 1994; Cuddy et al., 2007; Frijda, 2010). Further, research on emotions in behavioral economics has demonstrated that negative emotions, particularly anger enhances the decision to punish in economic exchanges (e.g., Scherer, 2005). In Experiment 2, we measured Anger toward the CSs, to investigate the role of this expressed emotion during aversive learning and the decision to administer shocks during the Test phase. As expected, our results show that Anger toward CSs paired with shocks and general aggressive traits increased the likelihood of administrating shocks to the aversively reinforced faces (i.e., CS+), but not to the non-aversively reinforced face (i.e, CS−). Examination of the relationship between aggressive trait, trial-by-trial anger and administration of shocks revealed that trait aggression mediated the impact of trial-by-trial anger towards the co-players on administration of shocks to the co-players. This suggests that anger and aggressive traits do not alone trigger aggressive behavior, at least not in our experimental model. However, following the receipt of punishment, trait and state anger are more likely to be expressed through retaliatory behaviors. These findings are in line with theories of aggression describing an interaction between person and situational factors influencing aggressive behaviors (Anderson and Bushman, 2001). Pointing toward a potential brain mechanism of this interaction, a recent study has shown a link between personality trait aggression and increased brain activity (e.g., superior temporal sulcus) when observing an aggressor and a victim interact (Van Den Stock et al., 2015). Furthermore, anger and aggressive traits are sensitive to the strength of the aversive event, that is, participants are angrier with the CS++ as compared to CS+. Another critical factor predicting aggressive behavior is expectancy of punishment. According to social learning theory, behavior is a function of expected probability of occurrence of a particular reinforcement (expectancy) and the degree of preference attached to that reinforcement (reinforcement value; Rotter, 1954); for example, someone who expects aggression to result in outcomes such as rewards (e.g., peer respect) will be more aggressive than someone who does not hold similar outcome expectations. Our results demonstrate that expectancy of receiving punishment predicted administration of shocks to the co-player, which is in line with this reasoning, indicating that aggressing toward the co-player may have had a positive value/outcome for the participant.

There are several potential explanations to why participants' retaliatory behaviors increased toward the aversively reinforced CSs. One possible explanation is that participants felt unfairly/unjustly treated, and that punishing the other person would “balance” the situation out (i.e., retribution). A second explanation is that participants attempted to alter the future behavior of the norm transgressor (co-player) by teaching him/her that acting unfairly does not pay (i.e., deterrence). Both retributive and deterrence are known to motivate human punishment (Crockett et al., 2014). Finally, a third reason to punish could simply be to harm the other person out of spite (de Quervain et al., 2004) because anger-induced punishment seems to give pleasure to the punisher (Berkowitz, 1993).

Anger may not be the only explanation for the decision to punish. A possible limitation in our paradigm is that the responder may, for instance, administer shocks because he or she feels the need to comply with what he or she believes is the appropriate behavior in the laboratory (Zizzo, 2010). However, participants had the option to give zero shocks, or remove the CS, which are two non-aggressive options, that argues against the explanation that participants wanted to comply with the experimental demand to administer shocks. In the post-experimental interviews, participants reported retributive motives for administering shocks, and none of the participants attributed their behavior to experimental demands. Furthermore, our results demonstrate that participants avoided CS− the most by choosing to remove this image rather than giving (0–2) shocks. Participants chose to remove the image of the CS++ the least. This indicates that, when given the opportunity to “interact” with the CS+'s participants opted for the options of giving shocks rather than removing the image (e.g., avoidance option) or giving zero shocks. As with most experimental tests of aggression/anti-social behaviors specifically, and experimental models of complex phenomena in general, the external validity (i.e., probability to generalize to situations outside the laboratory) is limited (see Tedeschi and Bond, 2001). However, others have argued that there is direct and indirect support for aggression paradigms in many research domains (e.g., Anderson and Bushman, 2001). We therefore believe that our findings have important potential application in terms of those circumstances in which an individual use previous aversive experiences to act retaliatory toward others. Finally, our design did not allow us to draw any conclusions about possible interactions between target and participant gender we hope that future studies can address how gender mismatch influence retaliatory behavior.

Our two experiments show the importance of aversive learning in social interactive behavior, and show how aggressive traits do not by themselves explain aggressive behavior. Rather, aggressive traits together with aversive learning experiences influence the decision to aggress toward others. In other words, depending on the individual's personality, the perception of being punished initiates retaliation that continues as long as expectation of being punished or anger at the punisher is high. Our findings have potential ramifications for important social phenomena, such as cycles of self-reinforced retaliatory behaviors. Further research providing insights into the processes underlying these types of behaviors, or detailing the boundary conditions under which it will occur, may prove to be valuable in developing strategies to prevent anti-social behaviors.

## Author contributions

TM, BL, and AO designed the experiment; TM performed the experiments; TM, BL analyzed the data; TM, BL; and AO wrote the paper.

### Conflict of interest statement

The authors declare that the research was conducted in the absence of any commercial or financial relationships that could be construed as a potential conflict of interest.
