# Supplementary material for: Aversive Learning and Trait Aggression Influence Retaliatory Behavior
Source: Front Psychol. 2016 Jun 8;7:833. doi: 10.3389/fpsyg.2016.00833 (PMC4896936; doi:10.3389/fpsyg.2016.00833)
Supplement: Supplementary file 1 [file Presentation1.pdf]

Supplementary materials

**Non-parametric testing:**

Experiment 1, SCR

In Experiment 1, the Shapiro-Wilks test was used to test for normality of the SCRs and revealed that the SCRs were not normally distributed ($W= .97$, $p= .048$) (normal skewness ($p= .29$)). We re-analyzed SCRs to CS++, CS+ and CS− using Friedman's test with SCRs to (CS++, CS+, CS−) as within-subjects variable. Friedman's test revealed a significant main effect of SCR, $\chi 2$ (2) = 8.96, $p = .011$. Post-hoc analysis with Wilcoxon signed-rank test was conducted, and revealed a significant difference between CS+ and CS- ($Z=2.13$, $p=0.033$), a trending difference between CS++ and CS- ($Z=1.89$, $p=0.059$), and a non-significant difference between CS++ and CS+ ($Z= .55$, $p= .584$).

Experiment 1, administered shocks

In Experiment 1, the Shapiro-Wilks test was used to test for normality of the administration of shocks measure and revealed that they were not normally distributed ($W= .09$, $p= .003$), (normal skewness ($p= .24$)). We re-analyzed administration of shocks to CS++, CS+, and CS− using Friedman's test with Shocks to (CS++, CS+, CS−) as within-subjects variable. Friedman's test reveal a significant main effect of CS, $\chi 2$ (2) = 21.14, $p = .000026$). Post-hoc analysis with Wilcoxon signed-rank test was conducted, and revealed significant differences between CS+ and CS- ($Z= 3.07$, $p= .002$), CS++ and CS- ($Z= 3.65$, $p= .00026$), and CS++ and CS+ ($Z= 3.17$, $p= .002$).

Experiment 2, SCR

In Experiment 2, the Shapiro-Wilks test was used to test for normality of the SCRs and revealed that the SCRs were not normally distributed ($W= .93$, $p= .00027$), (normal skewness ($p= .26$)). We re-analyzed SCRs to CS++, CS+ and CS− using Friedman's test with SCRs to (CS++, CS+, CS−) as within-subjects variable. Friedman's test revealed a significant main effect of SCR, $\chi 2$ (2) = 15.25, $p = .00049$. Post-hoc analysis with Wilcoxon signed-rank test was conducted, and revealed significant differences between CS+ and CS- ($Z= 3.34$, $p= .001$), CS++ and CS- ($Z= 2.08$, $p= .038$), but no significant difference between and CS++ and CS+ ($Z= 1.49$, $p= .14$).

Experiment 2, administered shocks

In Experiment 2, the Shapiro-Wilks test was used to test for normality of the administration of shocks measure and revealed that they were not normally distributed ($W= .88$, $p< .0001$), (normal skewness ($p= .25$)). We re-analyzed administration of shocks to CS++, CS+ and CS− using Friedman's test with Shocks to (CS++, CS+, CS−) as within-subjects variable. Friedman's test reveal a significant main effect of CS, $\chi 2$ (2) = 33.64, $p < .00001$). Post-hoc analysis with Wilcoxon signed-rank test was conducted, and revealed significant differences between CS+ and CS- ($Z= 4.18$, $p< .0001$), CS++ and CS- ($Z= 4.66$, $p< .0001$), and CS++ and CS+ ($Z= 2.29$, $p= .022$).

Experiment 2, expectancy of shocks ratings

In Experiment 2, the Shapiro-Wilks test was used to test for normality of expectancy to receive a shock measure and revealed that they were not normally distributed ($W= .93$, $p< .0001$), (skewness ($p= .25$)). We re-analyzed administration of shocks to CS++, CS+ and CS− using Friedman's test with expectancy of receiving shocks to (CS++, CS+, CS−) as within-subjects variable. Friedman's test revealed a significant main effect of CS, $\chi 2$ (2) = 37.3, $p< .00001$). Post-hoc analysis with Wilcoxon signed-rank test was conducted, and revealed significant differences between CS+ and CS- ($Z= 4.41$, $p< .0001$), CS++ and CS- ($Z= 4.78$, $p< .00001$), and CS++ and CS+ ($Z= 2.83$, $p= .005$).

Experiment 2, trial-by-trial anger ratings

In Experiment 2, the Shapiro-Wilks test was used to test for normality of trial-by-trial anger ratings and revealed that they were not normally distributed ($W= .89$, $p< .00001$), (normal skewness ($p= .25$)). We re-analyzed anger ratings to CS++, CS+ and CS− using Friedman's test anger to (CS++, CS+, CS−) as within-subjects variable. Friedman's test reveal a significant main effect of CS, $\chi 2$ (2) = 50.95, $p< .00001$). Post-hoc analysis with Wilcoxon signed-rank test was conducted, and revealed significant differences between CS+ and CS- ($Z=4.86$, $p< .0001$), CS++ and CS- ($Z=4.86$, $p< .0001$), and CS++ and CS+ ($Z=2.51$, $p= .012$).
